# Supplementary material for: A Likelihood Approach for Real-Time Calibration of Stochastic Compartmental Epidemic Models
Source: PLoS Comput Biol. 2017 Jan 17;13(1):e1005257. doi: 10.1371/journal.pcbi.1005257 (PMC5240920; doi:10.1371/journal.pcbi.1005257)
Supplement: S1 File — (TAR.GZ) [file pcbi.1005257.s014.tar.gz › HSPH_Online-SI-Revision/MSS10/n100-severe/n100-severe_NEW_table0.pdf]

| | | | | | | | | | | | | | | | | | | | | | | | | | | | |
|---|---|---|---|---|---|---|---|---|---|---|---|---|---|---|---|---|---|---|---|---|---|---|---|---|---|---|---|
| 1 | R0 | {1.65278, 9.06793, 0.1} | {1.454, 5.65591, 3.} | {{1.454, 5.65591, 3.}, {1.454, 5.65591, 3.}} | {12.0269, 37.6273, 2900.} | inf | 9005. | 4988.6 | {4838.68, 5122.4} | 44.6019 | 0.25663 | newly | 6768. | 5724.68 | {5532., 5878.} | 15.4155 | 0.0728362 | Reff | 0.958398 | 0.901531 | {0.899049, 0.903664} | 5.93353 | ar | 66 342. | 54 872.2 | {54 204., 55 552.} | 17.289 |
| 2 | R0 | {1.4321, 3.80644, 0.1} | {1.4498, 4.01058, 0.} | {{1.44762, 4.00571, 0.}, {1.44762, 4.00571, 0.}} | {1.23603, 5.36307, 100.} | inf | 4783. | 5046.26 | {4811.66, 5250.09} | 5.60136 | 0.0234648 | newly | 7700. | 7731.75 | {7393., 8067.} | 2.67571 | 0.0115697 | Reff | 0.875729 | 0.882733 | {0.878598, 0.885913} | 0.79975 | ar | 54 217. | 55 114.3 | {54 338., 55 756.} | 1.71963 |
| 3 | R0 | {1.68388, 4.76028, 0.1} | {1.64953, 4.30394, 2.} | {{1.64953, 4.30394, 2.}, {1.64953, 4.30394, 2.}} | {2.03981, 9.58634, 1900.} | inf | 8327. | 7335.71 | {7114.19, 7518.95} | 11.9045 | 0.0551564 | newly | 10 200. | 9727.42 | {9459., 9975.} | 4.64667 | 0.0207678 | Reff | 0.78489 | 0.785231 | {0.781812, 0.788705} | 0.276284 | ar | 68 299. | 66 651.8 | {66 133., 67 083.} | 2.41172 |
| 4 | R0 | {1.66014, 6.95383, 1.} | {1.5006, 4.69146, 2.41} | {{1.44762, 4.00571, 0.}, {1.54465, 5.30296, 4.}} | {9.61017, 32.5341, 197.} | inf | 9062. | 5832.03 | {4739.72, 6783.68} | 35.643 | 0.194905 | newly | 8732. | 7856.42 | {7343., 8340.} | 10.0273 | 0.0464136 | Reff | 0.943906 | 0.901409 | {0.883018, 0.914226} | 4.50228 | ar | 66 820. | 58 004. | {54 501., 60 897.} | 13.1937 |
| 5 | R0 | {1.69984, 11.2038, 1.} | {1.44485, 6.00596, 0.} | {{1.44485, 6.00596, 0.}, {1.44485, 6.00596, 0.}} | {15.001, 46.3935, 100.} | inf | 9747. | 4683.57 | {4559.38, 4799.49} | 51.9486 | 0.318386 | newly | 5980. | 5051.04 | {4884., 5177.} | 15.5344 | 0.0734326 | Reff | 0.958166 | 0.88759 | {0.885833, 0.889294} | 7.36568 | ar | 68 462. | 54 154.7 | {53 582., 54 811.} | 20.8982 |
| 6 | R0 | {1.65876, 6.66217, 1.} | {1.46551, 4.23546, 0.79} | {{1.44762, 4.00571, 0.}, {1.50982, 4.79544, 3.}} | {11.6502, 36.4251, 133.} | inf | 9053. | 5540.01 | {4993.44, 6599.21} | 38.8047 | 0.216194 | newly | 9118. | 8398.94 | {7977., 8921.} | 7.92498 | 0.0362512 | Reff | 1.0042 | 0.938475 | {0.928876, 0.957858} | 6.54469 | ar | 67 105. | 55 946.7 | {54 131., 60 056.} | 16.6282 |
| 7 | R0 | {1.69966, 5.89996, 0.1} | {1.78724, 6.63253, 2.} | {{1.78724, 6.63253, 2.}, {1.78724, 6.63253, 2.}} | {5.15266, 12.4165, 1900.} | inf | 10 219. | 11 509.4 | {11 316.8, 11 732.3} | 12.6277 | 0.051597 | newly | 11 408. | 11 488.7 | {11 257., 11 688.} | 1.36764 | 0.00590061 | Reff | 0.93415 | 0.959221 | {0.955186, 0.962585} | 2.6838 | ar | 69 396. | 72 784.1 | {72 295., 73 205.} | 4.88228 |
| 8 | R0 | {1.53428, 6.3734, 0.1} | {1.41632, 4.94998, 2.01} | {{1.41571, 4.94504, 2.}, {1.41571, 4.94504, 2.}} | {7.68817, 22.3337, 1910.} | inf | 6765. | 4813.37 | {4604.53, 4989.76} | 28.8489 | 0.148119 | newly | 7091. | 6341. | {6091., 6597.} | 10.5768 | 0.0487769 | Reff | 0.973639 | 0.926419 | {0.92353, 0.928984} | 4.84978 | ar | 59 855. | 52 799.2 | {52 093., 53 465.} | 11.7882 |
| 9 | R0 | {1.58939, 3.48608, 0.1} | {1.40843, 2.14634, 0.35} | {{1.39162, 1.89883, 0.}, {1.43965, 2.60601, 1.}} | {11.3852, 38.431, 380.} | inf | 7643. | 4269.92 | {3291.88, 5583.54} | 44.1329 | 0.262121 | newly | 13 814. | 10 876.6 | {9424., 12 378.} | 21.2636 | 0.105936 | Reff | 0.959229 | 0.897335 | {0.890366, 0.903636} | 6.45247 | ar | 62 575. | 51 566.7 | {48 484., 55 686.} | 17.5922 |
| 10 | R0 | {1.58528, 5.5593, 0.1} | {1.46577, 4.2329, 0.9} | {{1.44762, 4.00571, 0.}, {1.50982, 4.79544, 3.}} | {7.53898, 23.8592, 940.} | inf | 7290. | 5257.14 | {4710.36, 6025.21} | 27.8857 | 0.144026 | newly | 8668. | 7736.85 | {7284., 8122.} | 10.7712 | 0.0498908 | Reff | 0.933207 | 0.892517 | {0.881893, 0.908734} | 4.36026 | ar | 63 039. | 55 935.8 | {54 115., 58 806.} | 11.268 |
| 11 | R0 | {1.58305, 4.62721, 0.1} | {1.40859, 2.56421, 3.55} | {{1.4031, 2.55684, 1.}, {1.43965, 2.60601, 4.}} | {11.0207, 44.584, 3450.} | inf | 7294. | 3704.67 | {3367.29, 3995.83} | 49.2094 | 0.295195 | newly | 9915. | 8024.45 | {7286., 8637.} | 19.0676 | 0.0927004 | Reff | 0.910428 | 0.860651 | {0.853216, 0.877099} | 5.46738 | ar | 63 222. | 51 259.5 | {50 130., 52 097.} | 18.9215 |
| 12 | R0 | {1.70234, 9.34626, 0.1} | {1.47651, 5.4396, 3.} | {{1.47651, 5.4396, 3.}, {1.47651, 5.4396, 3.}} | {13.2659, 41.7992, 2900.} | inf | 9412. | 5294.91 | {5147.32, 5448.99} | 43.743 | 0.249945 | newly | 6925. | 6313.27 | {6105., 6491.} | 8.83365 | 0.0402946 | Reff | 0.976973 | 0.908158 | {0.905809, 0.910269} | 7.04368 | ar | 68 213. | 56 251.1 | {55 493., 56 923.} | 17.5361 |
| 13 | R0 | {1.68556, 9.74954, 1.} | {1.61188, 8.7843, 3.} | {{1.61188, 8.7843, 3.}, {1.61188, 8.7843, 3.}} | {4.37134, 9.90041, 200.} | inf | 9667. | 7861.73 | {7700.48, 8011.59} | 18.6746 | 0.0898203 | newly | 6771. | 6003.48 | {5869., 6130.} | 11.3354 | 0.0523169 | Reff | 0.938014 | 0.926109 | {0.923675, 0.928589} | 1.26917 | ar | 68 426. | 64 460.3 | {63 970., 64 952.} | 5.79563 |
| 14 | R0 | {1.59876, 9.78897, 1.} | {1.37383, 5.20813, 1.} | {{1.3618, 4.98002, 1.}, {1.40191, 5.7404, 1.}} | {14.0687, 46.7959, 0.} | inf | 7640. | 3913.14 | {3543.69, 4432.8} | 48.7808 | 0.292125 | newly | 5391. | 5020.26 | {4747., 5267.} | 6.92673 | 0.0315172 | Reff | 1.0474 | 0.951185 | {0.943681, 0.964565} | 9.18572 | ar | 63 553. | 48 811.3 | {47 212., 51 064.} | 23.1959 |
| 15 | R0 | {1.43577, 5.12819, 1.} | {1.43042, 4.90879, 3.01} | {{1.38889, 4.24222, 2.}, {1.47651, 5.4396, 4.}} | {2.95767, 12.1479, 201.} | inf | 5339. | 5044.49 | {4174.19, 5889.88} | 14.6163 | 0.0652934 | newly | 6966. | 6890.64 | {6482., 7420.} | 4.27419 | 0.0186924 | Reff | 0.97842 | 0.978523 | {0.959362, 1.00232} | 1.80657 | ar | 53 259. | 53 326.2 | {50 030., 57 023.} | 5.11576 |
| 16 | R0 | {1.58866, 8.73191, 1.} | {1.44485, 6.00596, 0.} | {{1.44485, 6.00596, 0.}, {1.44485, 6.00596, 0.}} | {9.05245, 31.2182, 100.} | inf | 7934. | 5366.09 | {5195.96, 5524.9} | 32.3659 | 0.169958 | newly | 6295. | 6133.16 | {5933., 6317.} | 2.91501 | 0.0129266 | Reff | 1.05624 | 0.997724 | {0.995404, 1.00016} | 5.53976 | ar | 64 349. | 54 531.5 | {53 866., 55 274.} | 15.2566 |
| 17 | R0 | {1.43012, 3.8163, 0.1} | {1.44762, 4.00571, 0.} | {{1.44762, 4.00571, 0.}, {1.44762, 4.00571, 0.}} | {1.22369, 4.96318, 100.} | inf | 5239. | 5279.77 | {5002.46, 5557.62} | 3.42191 | 0.0147552 | newly | 8949. | 8798.1 | {8354., 9218.} | 3.20751 | 0.0141666 | Reff | 0.975428 | 0.986774 | {0.98255, 0.990668} | 1.16319 | ar | 53 353. | 54 569.6 | {53 727., 55 325.} | 2.29987 |
| 18 | R0 | {1.47965, 5.16472, 0.1} | {1.47651, 5.4396, 3.} | {{1.47651, 5.4396, 3.}, {1.47651, 5.4396, 3.}} | {0.212245, 5.3223, 2900.} | inf | 5515. | 5923.97 | {5788.93, 6066.28} | 7.41552 | 0.0309777 | newly | 7086. | 7176.48 | {6971., 7361.} | 2.12927 | 0.00913998 | Reff | 0.947154 | 0.939105 | {0.936992, 0.941078} | 0.849779 | ar | 56 086. | 56 999.3 | {56 364., 57 560.} | 1.64068 |
| 19 | R0 | {1.57191, 5.2314, 0.1} | {1.55618, 4.83753, 3.} | {{1.55618, 4.83753, 3.}, {1.55618, 4.83753, 3.}} | {1.00099, 7.52899, 2900.} | inf | 6751. | 6082.37 | {5867.64, 6264.75} | 9.90411 | 0.0454162 | newly | 7954. | 7633.65 | {7392., 7870.} | 4.11604 | 0.0183578 | Reff | 0.829937 | 0.832034 | {0.829168, 0.834917} | 0.305435 | ar | 63 119. | 61 388.7 | {60 827., 61 918.} | 2.74128 |
| 20 | R0 | {1.54435, 5.32911, 0.1} | {1.50493, 4.71843, 2.88} | {{1.49267, 4.47101, 3.}, {1.50982, 4.79544, 3.}} | {2.5524, 11.4592, 2788.} | inf | 6968. | 5937.97 | {5516.38, 6226.32} | 14.7823 | 0.070104 | newly | 8356. | 7924.91 | {7575., 8261.} | 5.1636 | 0.0232214 | Reff | 0.898704 | 0.891232 | {0.887663, 0.895902} | 0.831397 | ar | 61 870. | 58 581. | {57 600., 59 417.} | 5.316 |
| 21 | R0 | {1.57932, 4.90031, 1.} | {1.55393, 4.27931, 0.24} | {{1.53501, 4.02511, 0.}, {1.69756, 6.15907, 2.}} | {3.4048, 18.8375, 100.} | inf | 8141. | 6818.31 | {6171.62, 9765.99} | 21.6121 | 0.103234 | newly | 10 527. | 10 121.3 | {9722., 10 651.} | 4.45825 | 0.0198994 | Reff | 0.930156 | 0.935136 | {0.927022, 0.965496} | 0.741417 | ar | 63 595. | 60 970.5 | {59 259., 68 934.} | 6.25725 |
| 22 | R0 | {1.47044, 4.07694, 1.} | {1.36813, 2.75833, 3.09} | {{1.37363, 2.81014, 3.}, {1.37363, 2.81014, 3.}} | {6.95785, 32.3432, 209.} | inf | 5758. | 3704.26 | {3350., 4055.74} | 35.6675 | 0.19334 | newly | 9422. | 8437.4 | {7801., 9093.} | 10.5075 | 0.0489868 | Reff | 0.991724 | 0.95078 | {0.946749, 0.957625} | 4.12857 | ar | 55 895. | 48 047.4 | {46 159., 49 285.} | 14.04 |
| 23 | R0 | {1.64997, 3.34546, 0.1} | {1.65456, 3.37285, 0.} | {{1.65456, 3.37285, 0.}, {1.65456, 3.37285, 0.}} | {0.278209, 0.818631, 100.} | inf | 6829. | 6884.57 | {6583.73, 7153.89} | 2.84534 | 0.0122685 | newly | 10 882. | 10 743.1 | {10 308., 11 113.} | 2.5488 | 0.0112249 | Reff | 0.75727 | 0.758458 | {0.753908, 0.763348} | 0.434613 | ar | 66 404. | 66 842. | {66 115., 67 400.} | 0.826592 |
| 24 | R0 | {1.67405, 4.50254, 0.1} | {1.64953, 4.30394, 2.} | {{1.64953, 4.30394, 2.}, {1.64953, 4.30394, 2.}} | {1.46459, 4.41077, 1900.} | inf | 9359. | 8800.8 | {8470.97, 9057.66} | 5.97423 | 0.0268978 | newly | 12 907. | 12 616.5 | {12 234., 12 989.} | 2.56373 | 0.0113454 | Reff | 0.908708 | 0.904607 | {0.899636, 0.909335} | 0.517014 | ar | 68 017. | 66 819.7 | {66 191., 67 363.} | 1.76037 |
| 25 | R0 | {1.50663, 3.1286, 0.1} | {1.44865, 2.71207, 1.18} | {{1.43965, 2.60601, 1.}, {1.4804, 3.12918, 2.}} | {3.99907, 14.0801, 1080.} | inf | 5849. | 4987.36 | {4431.52, 5878.02} | 16.5883 | 0.0794593 | newly | 11 133. | 10 408.8 | {9564., 11 282.} | 7.44831 | 0.0340311 | Reff | 0.89994 | 0.877669 | {0.87121, 0.883841} | 2.51183 | ar | 58 823. | 54 977.2 | {53 379., 57 634.} | 6.80074 |
| 26 | R0 | {1.69822, 6.34992, 0.1} | {1.55327, 4.37961, 1.32} | {{1.53501, 4.02511, 0.}, {1.59034, 5.09935, 4.}} | {8.53547, 31.029, 1354.} | inf | 9988. | 6545.65 | {5827.57, 7741.49} | 34.4649 | 0.186662 | newly | 10 139. | 9200.73 | {8836., 9556.} | 9.25407 | 0.0423842 | Reff | 0.906629 | 0.882508 | {0.877729, 0.88756} | 2.66048 | ar | 69 816. | 61 165.6 | {59 230., 64 283.} | 12.3903 |
| 27 | R0 | {1.55869, 4.92988, 0.1} | {1.57874, 5.00723, 3.7} | {{1.55618, 4.83753, 3.}, {1.59034, 5.09935, 4.}} | {1.43513, 3.03739, 3600.} | inf | 7340. | 7498.4 | {7010.26, 7846.96} | 4.34694 | 0.0186124 | newly | 9500. | 9589.76 | {9230., 9896.} | 2.30842 | 0.00993696 | Reff | 0.908124 | 0.917255 | {0.908408, 0.923737} | 1.03327 | ar | 62 344. | 63 070.7 | {61 464., 64 003.} | 1.79546 |
| 28 | R0 | {1.46085, 6.06899, 0.1} | {1.41582, 4.9488, 2.03} | {{1.41571, 4.94504, 2.}, {1.41571, 4.94504, 2.}} | {3.08226, 18.4575, 1930.} | inf | 5497. | 4315.33 | {4125.13, 4483.7} | 21.4966 | 0.105381 | newly | 5894. | 5653.11 | {5383., 5866.} | 4.217 | 0.0189204 | Reff | 0.925463 | 0.913668 | {0.911214, 0.916206} | 1.27453 | ar | 55 484. | 51 847.6 | {51 078., 52 585.} | 6.55402 |
| 29 | R0 | {1.63017, 6.25164, 0.1} | {1.5443, 5.29788, 3.99} | {{1.54465, 5.30296, 4.}, {1.54465, 5.30296, 4.}} | {5.26741, 15.2561, 3890.} | inf | 8283. | 6729.45 | {6523.86, 6930.26} | 18.7558 | 0.0903449 | newly | 8506. | 7994.44 | {7760., 8230.} | 6.01411 | 0.0270429 | Reff | 0.896577 | 0.87334 | {0.870258, 0.876573} | 2.59172 | ar | 66 231. | 61 270.4 | {60 635., 61 865.} | 7.48982 |
| 30 | R0 | {1.45173, 5.68165, 0.1} | {1.41571, 4.94504, 2.} | {{1.41571, 4.94504, 2.}, {1.41571, 4.94504, 2.}} | {2.4809, 12.9648, 1900.} | inf | 5301. | 4671.96 | {4470.69, 4864.2} | 11.8664 | 0.055045 | newly | 6273. | 6210.54 | {5956., 6461.} | 2.52734 | 0.0110849 | Reff | 0.953496 | 0.938746 | {0.936008, 0.941555} | 1.54693 | ar | 55 280. | 52 372.8 | {51 620., 53 037.} | 5.25899 |
| 31 | R0 | {1.55808, 2.77316, 0.1} | {1.58672, 3.05492, 4.} | {{1.58672, 3.05492, 4.}, {1.58672, 3.05492, 4.}} | {1.83831, 10.1602, 3900.} | inf | 4924. | 5816.13 | {5530.93, 6059.93} | 18.1181 | 0.0719947 | newly | 9080. | 10 004.5 | {9509., 10 389.} | 10.1814 | 0.0418394 | Reff | 0.782265 | 0.78249 | {0.7784, 0.78692} | 0.347603 | ar | 60 940. | 63 015.6 | {62 390., 63 652.} | 3.40592 |
| 32 | R0 | {1.56797, 3.90327, 0.1} | {1.53392, 4.00719, 0.04} | {{1.53501, 4.02511, 0.}, {1.53501, 4.02511, 0.}} | {2.17183, 3.45559, 136.} | inf | 6996. | 7029.13 | {6780.55, 7306.7} | 2.75417 | 0.0120701 | newly | 11 191. | 10 893.1 | {10 543., 11 256.} | 3.09177 | 0.0137088 | Reff | 0.933679 | 0.912878 | {0.908739, 0.917143} | 2.22791 | ar | 62 228. | 60 990.6 | {60 386., 61 758.} | 1.99378 |
| 33 | R0 | {1.70027, 5.43261, 0.1} | {1.61691, 4.41442, 4.} | {{1.61691, 4.41442, 4.}, {1.61691, 4.41442, 4.}} | {4.90277, 18.7422, 3900.} | inf | 8804. | 7052.62 | {6847.51, 7260.54} | 19.893 | 0.0964482 | newly | 10 094. | 9322.52 | {9049., 9589.} | 7.64296 | 0.0346313 | Reff | 0.817558 | 0.805793 | {0.802246, 0.809028} | 1.43901 | ar | 68 798. | 65 053.1 | {64 563., 65 611.} | 5.44338 |
| 34 | R0 | {1.61668, 6.86192, 0.1} | {1.44762, 4.00571, 0.} | {{1.44762, 4.00571, 0.}, {1.44762, 4.00571, 0.}} | {10.4572, 41.6241, 100.} | inf | 8157. | 4541.22 | {4289.58, 4780.11} | 44.3273 | 0.254701 | newly | 7767. | 6996.62 | {6594., 7346.} | 9.91863 | 0.0457245 | Reff | 0.928411 | 0.883668 | {0.880172, 0.887087} | 4.81933 | ar | 64 693. | 54 102.7 | {53 221., 54 931.} | 16.3701 |
| 35 | R0 | {1.55955, 3.45071, 0.1} | {1.54098, 3.31039, 5.} | {{1.54098, 3.31039, 5.}, {1.54098, 3.31039, 5.}} | {1.19078, 4.06641, 4900.} | inf | 6759. | 6629.06 | {6338.31, 6914.81} | 3.23354 | 0.0143124 | newly | 11 360. | 11 595.1 | {11 178., 12 081.} | 3.11743 | 0.0133093 | Reff | 0.876124 | 0.867694 | {0.863188, 0.872103} | 0.971864 | ar | 61 783. | 61 131.8 | {60 432., 61 735.} | 1.18402 |
| 36 | R0 | {1.65068, 4.91307, 0.1} | {1.75904, 5.97256, 0.35} | {{1.74092, 5.83416, 0.}, {1.75359, 5.92488, 1.}} | {6.56465, 21.5647, 388.} | inf | 9077. | 11 102.4 | {10 568.9, 11 180.8} | 22.3141 | 0.0845917 | newly | 12 338. | 12 638.7 | {12 303., 12 918.} | 2.65691 | 0.0113325 | Reff | 0.997374 | 1.02561 | {1.01788, 1.02821} | 2.83129 | ar | 66 453. | 71 568.1 | {70 744., 71 876.} | 7.69732 |
| 37 | R0 | {1.46131, 3.06714, 0.1} | {1.44128, 2.62694, 1.04} | {{1.43965, 2.60601, 1.}, {1.43965, 2.60601, 1.}} | {1.47493, 14.5139, 940.} | inf | 5039. | 4240.39 | {3811.31, 4589.48} | 16.1172 | 0.0773593 | newly | 9251. | 8775.59 | {7963., 9485.} | 6.09956 | 0.0278509 | Reff | 0.841612 | 0.841573 | {0.836704, 0.847688} | 0.412787 | ar | 56 452. | 54 026.2 | {52 818., 54 955.} | 4.3997 |
| 38 | R0 | {1.71034, 5.2546, 0.1} | {1.61691, 4.41442, 4.} | {{1.61691, 4.41442, 4.}, {1.61691, 4.41442, 4.}} | {5.46267, 15.9895, 3900.} | inf | 9467. | 7476.09 | {7238.94, 7711.84} | 21.03 | 0.102672 | newly | 11 210. | 10 125.8 | {9774., 10 406.} | 9.67145 | 0.0442857 | Reff | 0.857787 | 0.84312 | {0.83922, 0.846687} | 1.70985 | ar | 69 816. | 64 928.8 | {64 396., 65 474.} | 7.00007 |
| 39 | R0 | {1.43565, 5.57989, 1.} | {1.41583, 4.94526, 2.03} | {{1.41571, 4.94504, 2.}, {1.41571, 4.94504, 2.}} | {1.40622, 11.4008, 103.} | inf | 5165. | 4656.86 | {4450.27, 4863.01} | 9.83809 | 0.0452605 | newly | 6041. | 6040. | {5769., 6306.} | 2.62473 | 0.0114072 | Reff | 0.90825 | 0.902901 | {0.899892, 0.905545} | 0.614645 | ar | 54 407. | 52 727.5 | {51 907., 53 571.} | 3.09118 |
| 40 | R0 | {1.40327, 4.00764, 0.1} | {1.36035, 3.22945, 3.} | {{1.36035, 3.22945, 3.}, {1.36035, 3.22945, 3.}} | {3.05867, 19.4176, 2900.} | inf | 4452. | 3580.26 | {3346., 3808.02} | 19.5808 | 0.0951828 | newly | 7170. | 6943.24 | {6488., 7368.} | 4.5325 | 0.0202905 | Reff | 0.942464 | 0.925496 | {0.922318, 0.928635} | 1.80041 | ar | 51 976. | 47 592. | {46 519., 48 565.} | 8.43474 |
| 41 | R0 | {1.63612, 4.26741, 0.1} | {1.56331, 3.72349, 2.} | {{1.56331, 3.72349, 2.}, {1.56331, 3.72349, 2.}} | {4.45034, 12.746, 1900.} | inf | 7141. | 6081.05 | {5837.06, 6346.2} | 14.8432 | 0.0700257 | newly | 9905. | 9339.53 | {8958., 9666.} | 5.84382 | 0.0263255 | Reff | 0.840131 | 0.819313 | {0.81503, 0.823098} | 2.47798 | ar | 65 029. | 61 895.4 | {61 284., 62 498.} | 4.81873 |
| 42 | R0 | {1.57426, 6.50271, 0.1} | {2.10265, 12.8455, 2.24} | {{1.85376, 9.90957, 2.}, {2.21411, 14.2212, 3.}} | {34.7093, 102.017, 2142.} | inf | 7623. | 16 585.5 | {12 797.4, 18 545.2} | 122.328 | 0.344146 | newly | 7814. | 8964.75 | {8878., 9234.} | 15.7004 | 0.0633814 | Reff | 0.952711 | 1.0757 | {1.02081, 1.10394} | 13.5058 | ar | 62 522. | 80 925.5 | {75 054., 84 718.} | 31.2537 |
| 43 | R0 | {1.37818, 5.37636, 0.1} | {1.36136, 4.97277, 1.01} | {{1.3618, 4.98002, 1.}, {1.3618, 4.98002, 1.}} | {1.22045, 7.5067, 910.} | inf | 4296. | 3869.1 | {3683.4, 4044.02} | 9.93718 | 0.0457556 | newly | 5382. | 5144.87 | {4906., 5361.} | 4.61817 | 0.0207487 | Reff | 0.94794 | 0.94218 | {0.93993, 0.944716} | 0.607614 | ar | 50 098. | 48 138.6 | {47 271., 49 017.} | 3.91123 |
| 44 | R0 | {1.66606, 4.92641, 0.1} | {1.74282, 5.48843, 0.16} | {{1.737, 5.43578, 0.}, {1.74592, 5.58477, 0.}} | {4.60751, 11.4083, 244.} | inf | 9374. | 10 764.4 | {10 397.3, 11 038.7} | 14.8321 | 0.0598235 | newly | 12 427. | 12 933.1 | {12 632., 13 221.} | 4.07862 | 0.0172932 | Reff | 0.958134 | 0.977993 | {0.97224, 0.983996} | 2.07265 | ar | 67 066. | 70 975.6 | {70 345., 71 320.} | 5.8295 |
| 45 | R0 | {1.623, 5.9449, 0.1} | {1.48207, 4.21275, 0.69} | {{1.47488, 4.06661, 0.}, {1.50982, 4.79544, 3.}} | {8.683, 29.1368, 746.} | inf | 8468. | 5772.82 | {5360.28, 6457.54} | 31.8278 | 0.167569 | newly | 9497. | 8817.73 | {8482., 9176.} | 7.18764 | 0.0326031 | Reff | 0.985843 | 0.940124 | {0.934293, 0.945277} | 4.63748 | ar | 64 554. | 56 962.2 | {55 825., 59 164.} | 11.7604 |
| 46 | R0 | {1.65235, 5.51234, 0.1} | {1.51565, 4.2954, 1.48} | {{1.47608, 3.83841, 0.}, {1.59034, 5.09935, 4.}} | {8.27292, 22.0766, 1498.} | inf | 8716. | 6582.45 | {5627.64, 7974.11} | 24.4785 | 0.126445 | newly | 10 647. | 9789.16 | {9257., 10 374.} | 8.10576 | 0.0371185 | Reff | 0.988634 | 0.938721 | {0.923296, 0.963332} | 5.04865 | ar | 66 509. | 59 312.8 | {56 403., 63 753.} | 10.8199 |
| 47 | R0 | {1.62138, 8.82208, 0.1} | {1.44485, 6.00596, 0.} | {{1.44485, 6.00596, 0.}, {1.44485, 6.00596, 0.}} | {10.8878, 31.9212, 100.} | inf | 8399. | 4901.27 | {4752.19, 5054.5} | 41.6446 | 0.234032 | newly | 6515. | 5270.9 | {5096., 5457.} | 19.0959 | 0.0921661 | Reff | 0.936704 | 0.885254 | {0.882962, 0.887376} | 5.49262 | ar | 65 598. | 54 673.6 | {53 939., 55 272.} | 16.6535 |
| 48 | R0 | {1.64073, 6.69261, 0.1} | {1.51412, 4.88729, 2.82} | {{1.44762, 4.00571, 0.}, {1.54465, 5.30296, 4.}} | {7.71646, 26.9748, 2770.} | inf | 8727. | 6201.44 | {4938.67, 6938.51} | 28.9396 | 0.15204 | newly | 8793. | 8210.42 | {7669., 8582.} | 6.71443 | 0.0305277 | Reff | 0.968375 | 0.931584 | {0.907456, 0.944165} | 3.79928 | ar | 66 438. | 58 815.8 | {54 405., 61 328.} | 11.4727 |
| 49 | R0 | {1.65556, 3.796, 0.1} | {1.58601, 3.0516, 4.01} | {{1.58672, 3.05492, 4.}, {1.58672, 3.05492, 4.}} | {4.2011, 19.61, 3910.} | inf | 8332. | 6551.57 | {6208.72, 6950.49} | 21.3685 | 0.104805 | newly | 12 770. | 11 841.4 | {11 278., 12 409.} | 7.27204 | 0.0330587 | Reff | 0.845809 | 0.838506 | {0.83241, 0.843487} | 0.883089 | ar | 66 575. | 62 887.7 | {62 151., 63 639.} | 5.53861 |
| 50 | R0 | {1.56828, 3.65713, 0.1} | {1.56331, 3.72349, 2.} | {{1.56331, 3.72349, 2.}, {1.56331, 3.72349, 2.}} | {0.31709, 1.81443, 1900.} | inf | 6216. | 6234.59 | {5941.37, 6483.94} | 2.68978 | 0.011666 | newly | 9733. | 9558.24 | {9131., 9907.} | 2.66187 | 0.0117653 | Reff | 0.821841 | 0.818945 | {0.814996, 0.823368} | 0.431557 | ar | 62 761. | 62 098.4 | {61 389., 62 716.} | 1.12815 |
